# Reply to ‘Trace N-glycans including sulphated species may originate from various plasma glycoproteins and not necessarily IgG’

**DOI:** 10.1038/s41467-018-05082-y

**Published:** 2018-07-25

**Authors:** Jing-Rong Wang, Wei-Na Gao, Rudolf Grimm, Shibo Jiang, Yong Liang, Hua Ye, Zhan-Guo Li, Lee-Fong Yau, Hao Huang, Ju Liu, Min Jiang, Qiong Meng, Tian-Tian Tong, Hai-Hui Huang, Stephanie Lee, Xing Zeng, Liang Liu, Zhi-Hong Jiang

**Affiliations:** 1 State Key Laboratory of Quality Research in Chinese Medicine, Macau University of Science and Technology, Avenida Wai Long, Taipa Macau, China; 2 Macau Institute for Applied Research in Medicine and Health, Macau University of Science and Technology, Avenida Wai Long, Taipa Macau, China; 3 Agilent Technologies, 5301 Stevens Creek Blvd, Santa Clara, CA 95051 USA; 4 Key Labortory of Medical Molecular Virology of Ministries of Education and Health, Basic Medical College, Fudan University, Shanghai, 200032 China; 5 Lindsley F. Kimball Research Institute, New York Blood Center, New York, NY 10065 USA; 6 Faculty of Information Technology, Macau University of Science and Technology, Avenida Wai Long, Taipa Macau, China; 7 Department of Rheumatology and Immunology, Peking University People’s Hospital, 11 Xizhimen South Street, Beijing, 100044 China; 8 Division of Rheumatology, Jiujiang First People’s Hospital, Taling North Road 48, Jiujiang, 332000 China; 9 Agilent Technologies Hong Kong Ltd., Suite 2603, 26/F, AXA Tower, Landmark East, Kwun Tong, Hong Kong China; 10 Guangdong Provincial Hospital of Chinese Medicine, Dade Road 111, Guangzhou, 510120 China

## Introduction

The correspondence from Lauc et al.^1^ argues that a high level of H5N4S2 (abbreviated as Hex5HexNAc4NeuAc2 in our original article^2^, which has a combined relative abundance of 5.4 %) indicates contamination of other plasma glycoproteins in our immunoglobulin G (IgG) samples. Therefore, they speculate that many trace glycans identified in our study originated from other plasma glycoproteins, but not IgG. We are thankful for the interest in our work; however, we do not agree with this conclusion as it is inferred from a generalization of a “relative abundance-based indicator”, incorrect interpretation of our data, and more importantly, incomplete consideration of the intrinsic high sensitivity and specially improved detection sensitivity for acidic glycans of our method. Herein, we address the query point by point and provide additional experimental results in support of our published article.

Firstly, Lauc et al.^3–10^ claim that the relative abundance of H5N4S2 on IgG is below 1% in their >30,000 samples, and therefore a high level of H5N4S2 can be considered as an indicator of contamination. However, because relative abundance is merely calculated from the signal response without using standard, therefore can vary with the analytical method and instrument conditions, it is not suitable to generalize a relative abundance-based indicator to the studies of another lab (especially labs that use different analytical approaches). It is common to observe varied relative levels of glycans across studies of different labs. Taking H5N4S2 as an example, relative abundance varying from 0.57 to 10% has been reported^11,12^. As aforementioned, the relative level of H5N4S2 cannot be simply used to indicate contamination across studies. Another issue is that we captured IgG from serum by using protein A. Therefore, it is not applicable to speculate non-specific binding of denatured protein to protein G (a phenomenon often occurred during the purification of IgG from dried bloodstrains as mentioned by Lauc et al.) based on the observation of >5% H5N4S2 to our study.

**Fig. 1:**
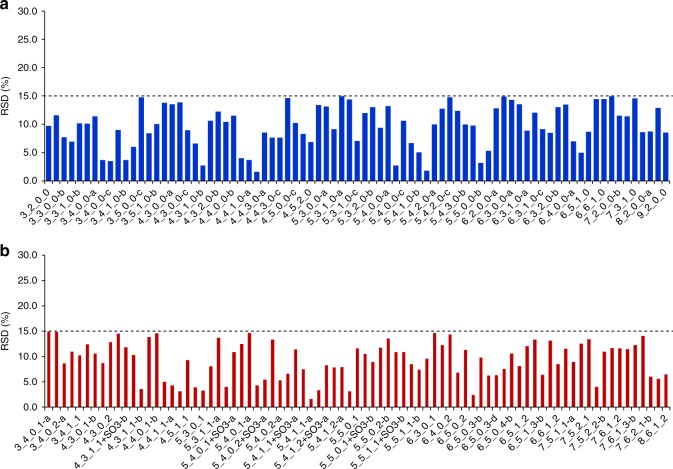
Quantification repeatability of N-glycans on HPLC Chip-QQQ MS. The repeatability was shown as the RSD (%) of ten replicate analyses. The RSD of relative abundance of both **a** neutral and **b** acidic N-glycans were all <15%

Of note, our method differs from that of Lauc’s lab in almost every step^3–10,12^, and was developed specifically to improve the detection of acidic glycans by: (1) on-chip enrichment (afford up to 25-fold signal gain); (2) a separated and optimized mobile phase for acidic glycans (enhanced the signal intensity of acidic glycans by approximately fivefold); and (3) optimized multiple reaction monitoring (MRM) parameters for quantification of each glycan species (further enhanced the detection of low-abundance species by ~1000-fold). Because of these improvements, the relative level of acidic glycans, especially those low-abundance acidic glycans, are commonly higher than the values reported by Lauc et al., while neutral glycans’ level are generally lower^3–10,12^. Hence, the relative high level of H5N4S2 in our study is mostly a result of the improved detection, not from other plasma glycoproteins. We would also clarify that the particularly high standard deviation mentioned by Lauc et al. refers to the deviation of the levels of ten healthy individuals (see Supplementary Data 4 of the original article^2^), but not the deviation of the calculated levels of the same sample. So the “particularly high standard deviation” indicated great individual difference, which is quite common and reasonable. It is not a correct judgement of varying levels of contaminating plasma glycoproteins on the basis of this deviation value, in fact that the repeatability of our analysis is quite good (with relative standard deviation (RSD) < 15%; Fig. 1).

**Fig. 2:**
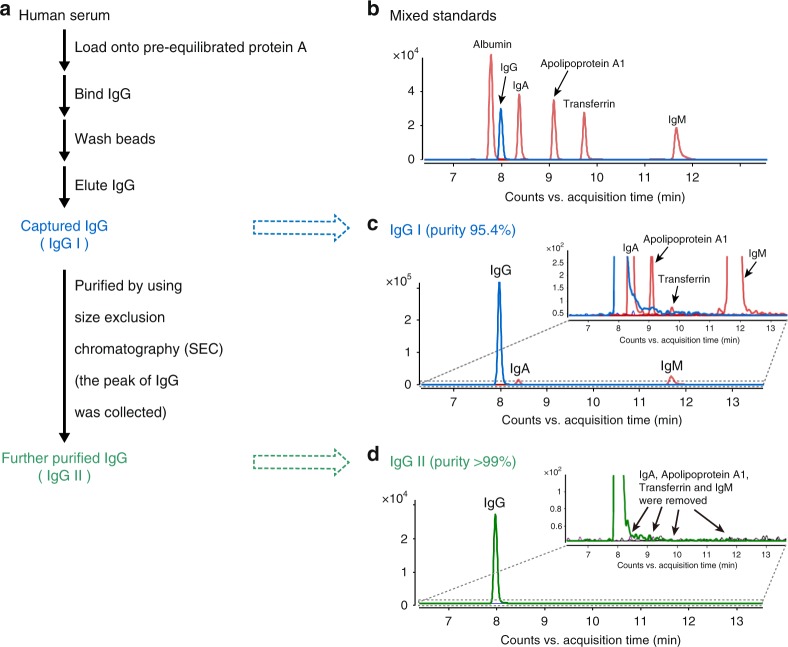
Capture and further purification of IgG from human serum. **a** A flow chart of capture and further purification of IgG from human serum. **b** MRM chromatograms of unique peptides of six glycoprotein standards (Albumin, IgG, IgA, Apolipoprotein A1, Transferrin, and IgM). **c, d** MRM chromatograms of unique peptides of IgG and other glycoproteins in captured IgG (IgG I) and further purified IgG (IgG II)

Secondly, we examined the purity of captured IgG in our original article by using two widely accepted methods, SDS-PAGE and size exclusion chromatography (SEC) HPLC, showing that the purity of IgG is >99% and 97.3%, respectively. In this reply, we further show the purity of IgG as 95.4% by using a more sensitive method, UHPLC-QQQ-MS detection of unique peptide in MRM mode, which is an advanced technique for consistent and precise quantification of targeted proteins^13,14^. In addition, IgM, IgA, and apolipoprotein A1 are identified as major impurities, and their contents are quantified as 2.35 ± 0.10%, 0.63 ± 0.04%, and <0.1%, respectively, in the captured IgG, based on the detection of each unique peptide in MRM mode. Notably, transferrin, the impurity suspected by Lauc et al. as a major origin of H5N4S2, is confirmed to be trace species (<0.01%) in our IgG samples (Fig. 2b, c). Subsequently, we performed quantitative analysis of N-glycans on commercial IgA and IgM standards (Sigma) and estimate that the theoretical glycan interference from IgA and IgM is <5% of corresponding N-glycans coming from the IgG samples.

Thirdly, we captured IgG (IgG I) from another pool of human serum following the method described in our original article, which is a well-accepted method for the capture of IgG^15,16^. Then we removed impurities of IgA, IgM, apolipoprotein A1, and transferrin by using SEC method to yield a further purified IgG sample (IgG II) (Fig. 2a). We performed glycomic analysis of IgG I and II by using the approach in our original article. As a result, all 185 N-glycan compositions reported in our original article are consistently identified on both IgG I and IgG II, although 12 trace glycan isomers are not detected due to the varied level among serum samples (we used another batch of serum sample in this reply). This result provides convincing evidence for the origin of >400 N-glycans from IgG, which are not from other plasma glycoproteins. In addition, we performed N-glycan analysis on purchased commercial human IgG (Sigma, purity >95%, no IgA and IgM were detected based on MRM detection of unique peptide), and a total of 410 N-glycans arising from 181 compositions are detected. Moreover, we identified 353 glycan structures arising from 172 compositions on rituximab, an antibody drug that does not contain any plasma glycoproteins. These results collectively demonstrate intrinsic structural diversity of the N-glycans on IgG.

On the basis of extensive examination of IgG impurities, glycan analysis of purified IgG and IgG samples from other origins (commercial IgG standard and antibody drug), we conclude that the glycans identified in our study do exist on IgG, but are not from other glycoproteins.

Because of technical limitations, till now only no more than 40 glycoforms have been identified for IgG by glycopeptide analysis. It should be noted that glycopeptides of H5N4S2 have been identified in human serum^17^ and recombinant IgGs^18^ in several glycopeptide studies. We have also developed a relatively sensitive method for glycopeptide analysis of IgG, which allows the detection of 72 glycopeptides (including the glycopeptide of H5N4S2)^19^. The evidence collectively showed that current glycopeptide analysis technique is far from providing evidence for comprehensive glycan analysis.

Finally, we would say that although lack of site-specific information, analysis of the released glycans by using PNGase F is the most common strategy for glycosylation analysis of glycoprotein (including IgG)^11,20^. Complementary glycopeptide analysis is still a great challenge to sensitively identify the glycans on glycoprotein (including IgG). We would investigate the more sensitive method of glycopeptide analysis to confirm the attachment of 444 glycans on IgGs in the future.

## Methods

### Further purification of captured IgG by using SEC method

An Agilent HPLC 1200 system was used for further purification of captured IgG. The captured IgG (IgG I) was purified on a Bio-Rad SEC column (Bio-Sil SEC 125-5, 300 × 7.8 mm, 5 µm), under an isocratic elution by 100 mM ammonium formate at a flow rate of 1 ml min^−1^. The detection wavelength was 280 nm. Based on gel filtration standard (Bio-Rad), the peak at 7.4 min was identified as IgG and collected as further purified IgG (IgG II).

### Analysis of purified IgG and other impurities by MRM

The standards of albumin and the dominant glycoproteins in plasma, including fibrinogen, α2-macroglobulin, transferrin, apolipoprotein A1, IgA, IgG, and IgM were purchased from Sigma. The unique peptide of each protein was selected and evaluated by blasting them against the Uniprot databases. The proteins were quantified by detecting MRM transitions of corresponding unique peptide on an Agilent 1290 Infinity UHPLC system coupled to an Agilent 6490 iFunnel QQQ MS in positive mode. The purity of IgG and the content of other impurities in the captured IgG was calculated by the standard curve plotted by the detection of standard at different concentrations.

### Data availability

The data that support the findings of the study are available from the corresponding author upon reasonable request.
